# Successful Use of Erlotinib in Treating Recurrent Thymic Carcinoma: A Case Report

**DOI:** 10.4021/wjon707w

**Published:** 2013-09-27

**Authors:** Lisa A. Brown

**Affiliations:** Clinical Research Department, Research Medical Center, 6420 Prospect Avenue, Suite T211, Kansas City, MO 64132, USA. Email: labm84@mail.umkc.edu

**Keywords:** Thymic, Carcinoma, Thymus, Cancer, Erlotinib, Recurrent

## Abstract

Thymic carcinomas are rare and aggressive tumors. Primary treatment for these tumors consists of surgical resection, followed by adjuvant radiation therapy or platinum based chemotherapy. Unfortunately these tumors often exhibit a high incidence of local recurrence and metastasis despite treatment. Of recent interest are new targeted therapies such as Tarceva^®^ (erlotinib), an epidermal growth factor receptor (EGFR) inhibitor, for treatment of recurrent thymic carcinoma. Unfortunately recent literature has shown little success with its use, except for a few isolated case reports. Here we present a unique case of progressive disease despite 4 cycles of cisplatin, doxorubicin, vincristine, and cyclophosphamide (ADOC) therapy, and subsequent treatment with 150mg erlotinib daily resulting in partial response at 6 months with tumor shrinking in size and resolution of many metastatic nodules.

## Introduction

Thymic carcinoma is a rare tumor that accounts for only 5% of thymic malignancies [1]. Due to its aggressive nature, 5 year survival rates have been reported in the range of 30-50% [2]. Even with modern therapy, median survival is only 2 years [3]. At time of diagnosis, over 60% of patients with thymic carcinomas have locally advanced disease or distant metastases, most common in lungs, pleura, and bones [1]. Furthermore, local tumor recurrence is common after undergoing primary treatment with surgical resection [1].

Extent of resection is predictive of the rate of recurrence [2]. Resection is often followed by adjuvant radiation therapy +/- chemotherapy. The commonly used chemotherapy regimens are currently carboplatin/paclitaxel, and cisplatin, doxorubicin, vincristine, and cyclophosphamide (ADOC) [2, 4]. In one study of 32 patients with stage III, classified as macroscopic invasion of tumor into neighboring organs with or without invasion of great vessels, or stage IV invasive thymoma, classified as including pleural or pericardial dissemination and lymph or hematogenous dissemination [2], ADOC therapy was shown to have a total response rate (partial and complete response) of 91%, with a complete remission rate of 47% [4].

Due to the rarity of these tumors, studying and performing clinical trials of different chemotherapeutic regimens has been difficult, but new therapies are starting to emerge. This includes molecular targeted therapies such as tyrosine kinase inhibitors (TKIs). One such TKI is erlotinib, an EGFR inhibitor. The rationale behind the use of erlotinib in thymic carcinomas is EGFR overexpression in these tumors causing uncontrolled cell proliferation and inhibition of apoptosis [5]. The use of an EGFR inhibitor like erlotinib in these tumors causes regression through anti-angiogenesis and apoptotic effects [3]. While EGFR is commonly overexpressed, activating mutations are rarely seen, which may account for the lack of consistent response with TKIs [6].

Erlotinib has successfully been used to treat non-small cell lung carcinoma and pancreatic carcinoma [7, 8]. Its effect on thymus tumors has been less impressive according to recent literature, although there are a few other isolated cases of success [9, 10]. It is typically used in the setting of progressive disease despite resection and platinum based chemotherapy. It had been previously studied to treat recurrent thymic carcinoma and invasive thymoma in combination with bevacizumab, but no tumor response was seen [11]. In a 2008 Italian case report, a patient with chemorefractory recurrent metastatic thymoma was found to have increased EGFR expression and was then started on erlotinib. After four months of therapy, she failed to achieve any response [12].

Our case presents the successful use of erlotinib post-resection, radiation therapy, and ADOC therapy in treating progressive recurrent thymic carcinoma. The patient has been on erlotinib for 6 months with metastatic tumors shrinking in size and resolution of many metastatic foci.

## Case Report

The patient is a 78-year-old African American male with a history of B2 predominant lymphocytic thymoma, stage IIIa, diagnosed in 2007. He originally presented with chest pain and was found to have a mediastinal mass. This mass was biopsied and diagnosed as a stage III widely invasive malignant thymoma with bronchial and vascular invasion. He underwent surgical resection of the mass, including part of the lung, and radiation therapy. Post-surgery, the patient experienced vocal cord paralysis and was treated with thyroplasty.

He was doing well and had no recurrent disease until April 2012, when he started having chest pain. Repeat computed tomography (CT) scan of the chest showed multiple new pleural nodules in the patient’s left hemithorax. Biopsy results were interpreted as recurrent thymic carcinoma. In May 2012, he was started on the ADOC chemotherapy regimen- intravenous (IV) cisplatin 50 mg/m^2^ and doxorubicin 40 mg on Day 1, 0.6 mg/m^2^ vincristine on day 2, and 700 mg/m^2^ cyclophosphamide on day 3, every 4 weeks. He also received continuous IV fluids, and on Day 5 received pegfilgastrim. He was started on sandostatin due to high chromogranin levels, and given dexamethasone and antiemetics prior to starting chemotherapy. The patient tolerated his first cycle of chemotherapy well with minimal side effects.

Repeat CT of the chest in June 2012 showed the patient was having a response to the chemotherapy with marked decrease in size of the metastatic disease, with one pleural nodularity decreasing from 7.3 × 1.8 cm to 5.0 × 1.3 cm. The patient completed three additional rounds of ADOC therapy for a total of four cycles, the last one being in August 2012. More therapy was not pursued due to adverse effects of fatigue and dizziness, and it was decided to observe the patient for 2 months.

In October 2012, PET/CT scan showed progression of the metastatic disease with pleural thickening and new bilateral lung nodules. Different treatment options were discussed, and in late October 2012 the patient was started on erlotinib 150 mg daily. Improvement was evident after a few months of treatment. PET/CT scan in December 2012 revealed that the small right sided lung nodules had resolved. Repeat PET/CT scan in April 2013 showed a positive response with significant improvement in the patient’s pleural metastases with a decrease in size and FDG uptake. Side effects of erlotinib experienced by the patient included diarrhea and a macular papular rash on the face and nose. He was started on loperamide for the diarrhea, and for the rash he was given cetaphil lotion, doxycycline 100 mg twice daily, and hydrocortisone 0.2% cream. As of April 2013, the patient continues to take erlotinib 150 mg daily and has no evidence of recurrent disease.

## Discussion

The use of erlotinib after 6 months produced a partial response in this patient with recurrent thymic carcinoma. The patient previously completed standard therapy recommendations of ADOC chemotherapy for his recurrent disease, which achieved a partial response. However after stopping this therapy due to adverse effects, the disease progressed further. Erlotinib therapy was started, and PET/CT scans 2 months later showed the resolution of small nodules in the right lung, and scans six months later showed significant improvement of the pleural metastases with marked decrease in FDG uptake. This case is limited, however, in the time of follow up. It has only been six months since starting erlotinib therapy, and thymic carcinomas are aggressive and have a high recurrence rate, so we cannot be sure how long this response will be sustained.

While the use of erlotinib in treating thymic malignancies has previously been relatively unsuccessful, there are a few other isolated cases of success reported in the literature. A Japanese case reported success in shrinking the size of a tumor in a 43-year-old woman with recurrent thymoma who had tried 8 other therapies unsuccessfully [9]. In Greece, the use of erlotinib in another 43-year-old woman with recurrent disease led to a partial response for 12 months. In the twelfth month, she worsened symptomatically and was found to have recurrent disease [10].

Larger studies have revealed less impressive results. Erlotinib has been previously studied in combination with bevacizumab to treat recurrent disease, but with limited activity [11]. Sixty percent of patients in this study achieved stable disease, and no partial or complete responses were seen. In a 2008 Italian case report, a patient with chemorefractory recurrent metastatic thymoma was found to have increased EGFR expression and was then started on erlotinib. After four months of therapy, she had still failed to achieve any response [12].

Multi-kinase inhibitors such as sunitinib and sorafenib have shown more consistent positive results. In one study, sunitinib therapy resulted in partial responses in 3 patients of 2 - 18 months, and stable disease in another. The success of sunitinib also was unrelated to mutations in EGFT, c-KIT, KRAS, and BRAF [3]. Sorafenib was used in a case of thymic carcinoma resistant to cisplatin-based chemotherapy, causing metastatic tumors and the primary tumor to shrink, and stable disease for 9 months [13].

### Conclusion

Targeted therapies, such as tyrosine kinase inhibitors, have been increasingly successful in the treatment of thymic malignancies. To the best of our knowledge, this is the third reported case of erlotinib causing a response in recurrent thymic malignancies. This case’s success suggests that targeted therapies would be beneficial as second-line choices after platinum based regimens. More specifically, erlotinib and EGFR inhibitors may have undiscovered potential at treating thymic malignancies that should be further explored with larger retrospective series and ultimately prospective data.
